# Supplementary material for: PRMT3 interacts with ALDH1A1 and regulates gene-expression by inhibiting retinoic acid signaling
Source: Commun Biol. 2021 Jan 25;4:109. doi: 10.1038/s42003-020-01644-3 (PMC7835222; doi:10.1038/s42003-020-01644-3)
Supplement: Supplementary file 2 — Supplementary Information [file 42003_2020_1644_MOESM2_ESM.pdf]

Supplementary information for

PRMT3 interacts with ALDH1A1 and regulates gene-expression by inhibiting retinoic acid signaling

Mamta Verma[1#], Mohd. Imran K. Khan[1#], Rajashekar Varma Kadumuri[2], Baskar Chakrapani[1], Sharad Awasthi[1], Arun Mahesh[1], Gayathri Govindaraju[3], Pavithra L. Chavali[4], Arumugam Rajavelu[3], Sreenivas Chavali[2]* & Arunkumar Dhayalan[1]*

[1]Department of Biotechnology, Pondicherry University, Puducherry - 605 014, India

[2]Department of Biology, Indian Institute of Science Education and Research (IISER) Tirupati, Tirupati- 517507, India

[3]Interdisciplinary Biology, Rajiv Gandhi Centre for Biotechnology, Trivandrum- 695 014, India

[4]CSIR-Centre for Cellular & Molecular Biology, Hyderabad - 500 007, India

[#] Equal contribution

[*]Correspondence to: Tel: +91 877 2500905; E-mail: schavali@iisertirupati.ac.in
Tel: +91 413 2654789; E-mail: arun.dbt@pondiuni.edu.in

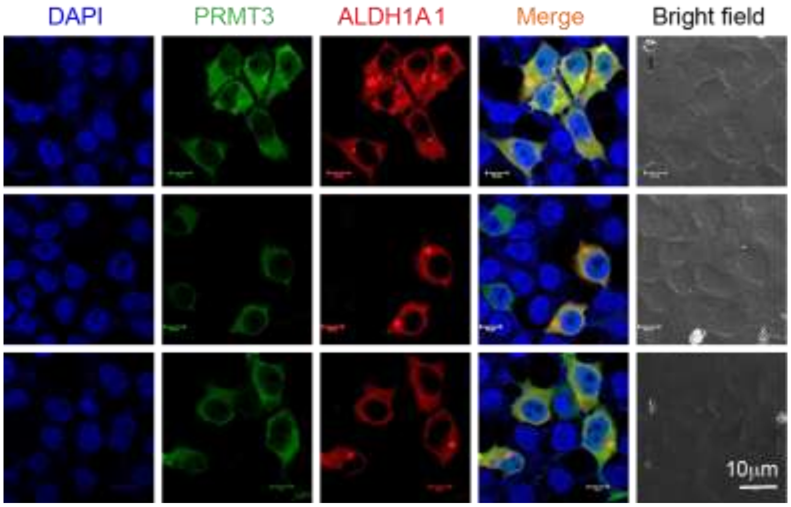

Supplementary Fig. 1. PRMT3 co-localizes with ALDH1A1.

The GFP-tagged ALDH1A1 and the DsRed-tagged PRMT3 were co-transfected in HEK293 cells. About 48 hours post-transfection, the cells were fixed and the images were captured using confocal laser-scanning microscopy. Representative images depicting the co-localization are shown.

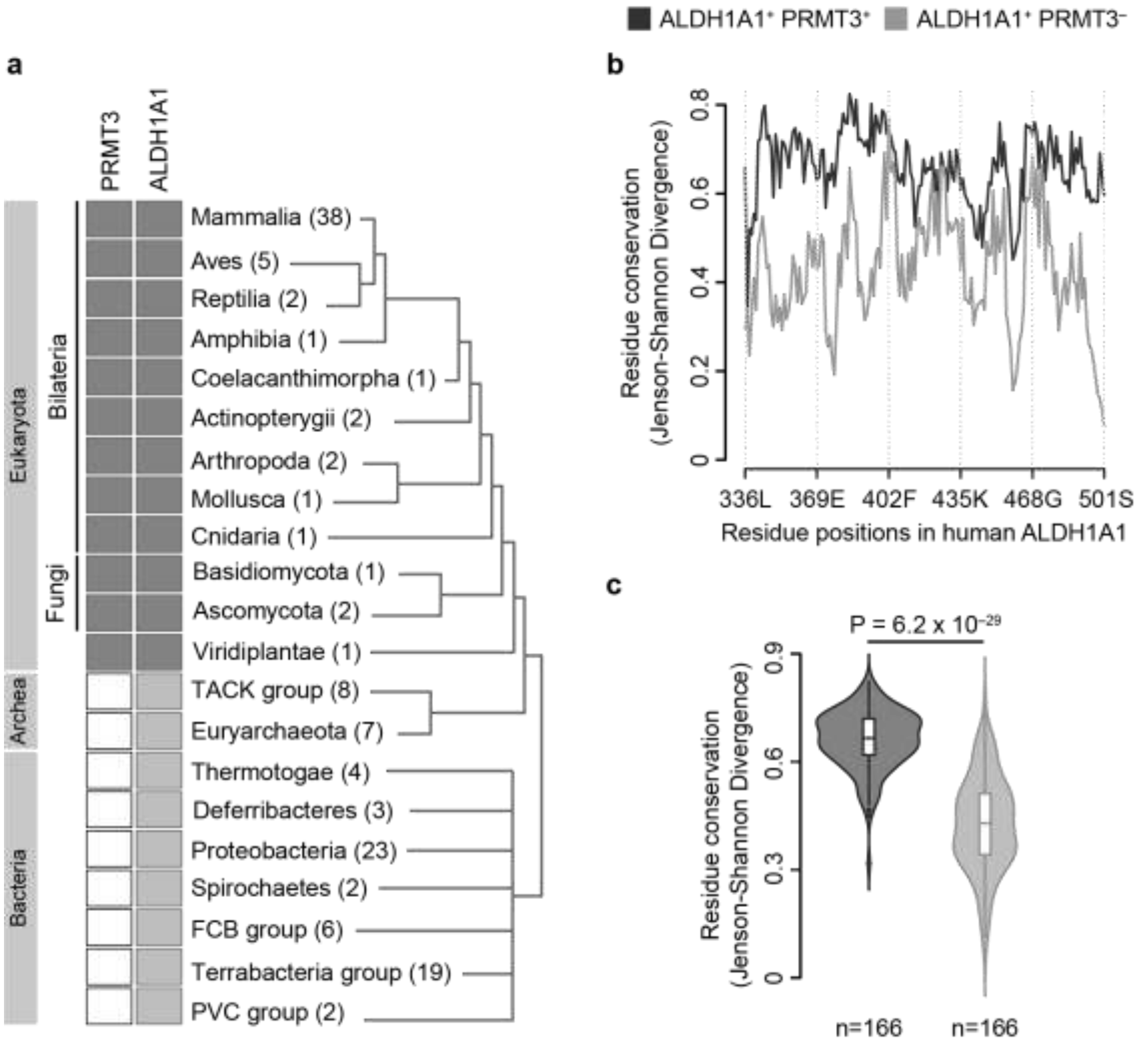

Supplementary Fig. 2. Conservation of residues in the C-terminal region of ALDH1A1 shows correlation with the evolution of PRMT3.

(**a**) Sequences of PRMT3 and ALDH1A1 were analyzed in diverse species spanning different phylogenetic classes of cellular organisms. Dark grey cells depict the organisms, which contain PRMT3 and ALDH1A1, while the light grey cells indicate organisms that contain ALDH1A1and not PRMT3. Numbers in parenthesis indicate the number of organisms in each class for which ALDH1A1 and/or PRMT3 sequences are available. The cladogram was reconstructed based on taxonomic classifications and the branch lengths do not represent any evolutionary attribute. (**b**) Jenson-Shannon Divergence (JSD) estimates for each residue for the human ALDH1A1 C-terminal region residues (336-501) that form the PRMT3-ALDH1A1 interaction interface. JSD was calculated for each residue in two sets of multiple sequence alignments (MSA) containing orthologs from organisms in which (i) both ALDH1A1 and PRMT3 were present (dark grey line; $ALDH1A1^{+}PRMT3^{+}$) and (ii) ALDH1A1 was present but PRMT3 was absent (light grey line;

ALDH1A1^{+}PRMT3$^{–}$). (**c**) Violin plot showing distribution of JSD estimates for human ALDH1A1 residues in MSA with ALDH1A1^{+}PRMT3^{+} orthologs and ALDH1A1^{+}PRMT3$^{–}$ orthologs. The box in the middle of each of distribution shows the inter-quartile range, with the horizontal line representing the median. The vertical lines outside the box represent 95% confidence interval. The dark/light grey outline provides the kernel density estimation of the distribution of the data. Statistical significance (depicted by P-value) was assessed using Wilcoxon-matched pairs test.

The catalysis of aldehyde oxidation is an evolutionary conserved biochemical process and ALDH1A1 is a very important enzyme that catalyzes this reaction. Therefore, we hypothesized that the region of ALDH1A1 that facilitates interaction with PRMT3 should be more conserved in species with PRMT3, compared to those in which PRMT3 is absent. To examine this, we first obtained all orthologs for human PRMT3 and ALDH1A1 proteins from OMA orthology database [1]. We found one-to-one orthologs of PRMT3 across all phylogenetic classes of eukaryotes ranging from metazoans, choanoflagellates, fungi, stramenophiles and alveolates, but could not find orthologs in bacteria or archaea. This indicated that PRMT3 is primarily of eukaryotic origin. Being a ubiquitous enzyme involved in a fundamental pathway of life, we identified orthologs of ALDH1A1 across all kingdoms of cellular organisms, as expected. Using stringent selection criteria, we obtained the most likely one-to-one orthologs of ALDH1A1 spanning bacteria, archaea and eukaryotes (see Materials and Methods for details). We then retrieved two sets of orthologs of ALDH1A1 (i) from eukaryotes, which contained both PRMT3 and ALDH1A1 (ALDH1A1^{+}PRMT3^{+}, n=57) and (ii) from prokaryotes that did not contain PRMT3 (ALDH1A1^{+}PRMT3$^{–}$, n=74; Supplementary Fig. S2a). We then generated a multiple sequence alignment to obtain conservation profiles using these two classes of ALDH1A1 orthologs. There were about 20 lower eukaryotic species in which we could not detect the ortholog of human PRMT3.

To estimate the extent of conservation for each residue within the C-terminal region of human ALDH1A1 that forms the interface of PRMT3-ALDH1A1 interaction, we computed Jensen-Shannon Divergence (JSD) [2]. JSD is based on symbol frequencies with relative entropy in its definition and is a highly reliable method for scoring conservation profiles [3,4]. JSD derives the background amino acid frequencies for sites that are not under any evolutionary pressure. Columns of residues in an alignment with high divergence from the background distribution are considered to be under high evolutionary constraints and hence functionally important. We find that the residues in the C-terminal region of ALDH1A1 in species with PRMT3 have significantly higher JSD and hence are more conserved compared to those in species that lack PRMT3 (Supplementary Fig. S2b, S2c). Taken together, these findings indicate that the C-terminal region of ALDH1A1 that interacts with PRMT3 might have coevolved with PRMT3.

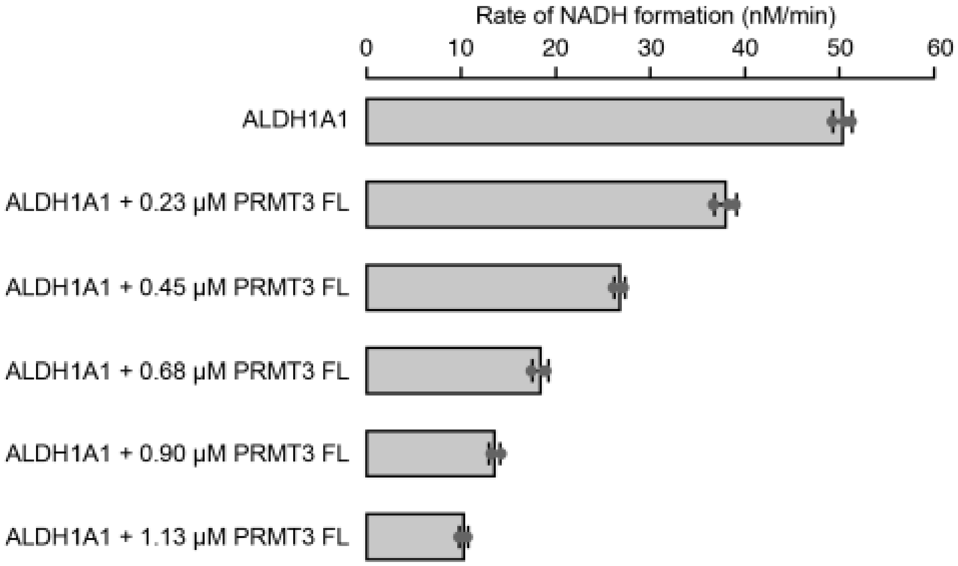

Supplementary Fig. 3. PRMT3 inhibits the ALDH1A1 activity in a concentration-dependent manner.

ALDH1A1 activity was measured without or with the increasing concentration of GST-tagged PRMT3 full length (FL) protein. The mean slopes of the three independent reactions were plotted as rate of the reactions against the concentration of PRMT3. Error bar indicates the standard deviations of the mean (Supplementary Data 1).

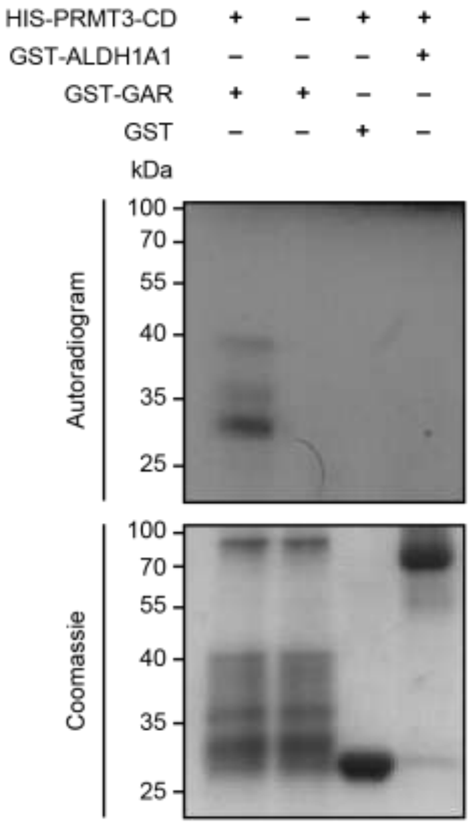

Supplementary Fig. 4. ALDH1A1 is not methylated by PRMT3.

Methylation assay was performed by incubating recombinant GST or GST-ALDH1A1 or GST-GAR proteins with or without His-tagged catalytic domain of PRMT3 and in the presence of radioactively labelled SAM. Methylation signals were quantified by autoradiography (upper panel). Proteins which were used in methylation assay were resolved in 12% SDS PAGE and stained with coomassie blue dye (lower panel) (Supplementary Fig. 10).

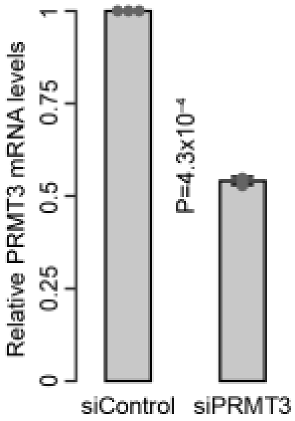

Supplementary Fig. 5. Efficiency of siRNA mediated knockdown of PRMT3.

HEK293 cells were transfected with control siRNA or PRMT3 siRNA and the mRNA levels of PRMT3 were quantified using qRT-PCR. PRMT3 mRNA levels were normalized to GAPDH expression and are presented relative to the control sample. Data are represented as mean of three independent experiments, with error bars representing standard deviation. Statistical significance was assessed by two tailed t-Test (Supplementary Data 1).

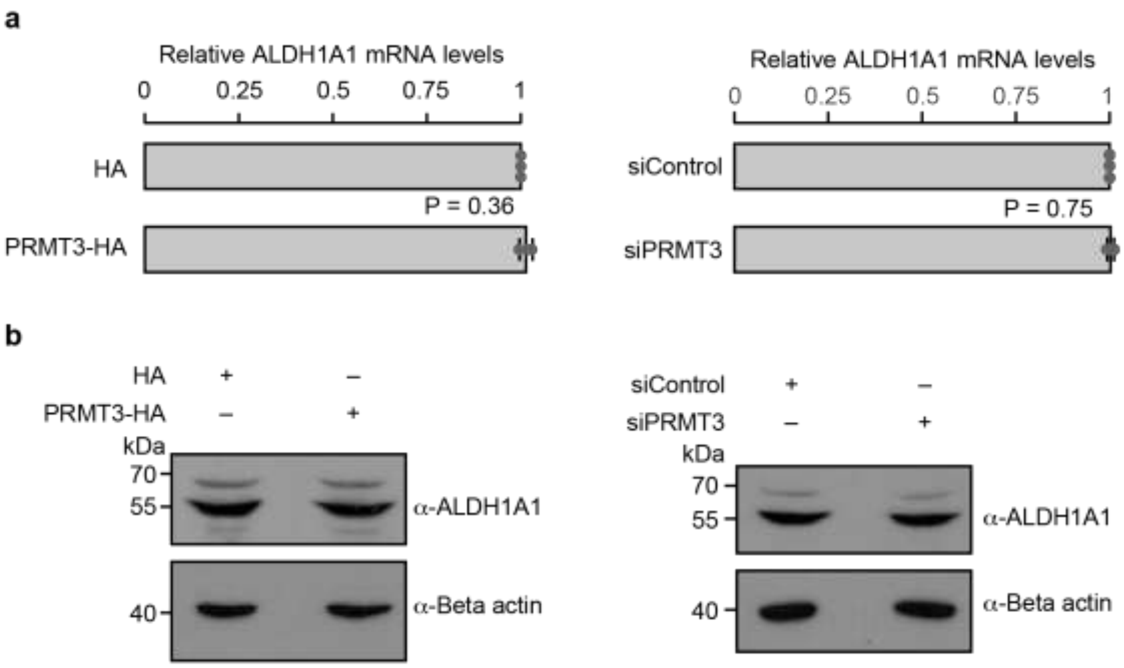

Supplementary Fig. 6. ALDH1A1 levels are not altered by the perturbation of PRMT3.

(a) HEK293 cells were transfected with HA vector or HA-PRMT3 construct or control siRNA or PRMT3 siRNA and the mRNA levels of ALDH1A1 were quantified using qRT-PCR. ALDH1A1 mRNA levels were normalized to GAPDH expression and are presented relative to the control sample. Data are represented as mean of three independent experiments, with error bars representing standard deviation. Statistical significance was assessed by two tailed t-Test. (Supplementary Data 1) (**b**) HEK293 cells were transfected with HA vector or HA-PRMT3 construct or control siRNA or PRMT3 siRNA and the protein levels of ALDH1A1 were quantified using immunoblotting with ALDH1A1 antibody (Supplementary Fig. 10).

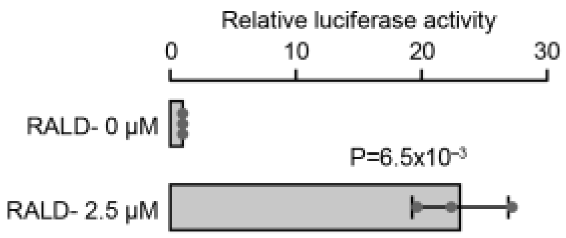

Supplementary Fig. 7. RALD treatment increases the luciferase expression of RARE-luciferase construct.

HEK293 cells were co-transfected with pGL3-RARE-luciferase and pRL-Renilla-luciferase reporter vector and treated with or without 2.5 µM of all trans-retinal (RALD). Luciferase reporter activities in the two conditions were quantified in these cells. The firefly luciferase activity was normalized to renilla luciferase activity and presented in the graph as relative to the control sample. The values in the graphs represent the mean of three independent experiments, with error bars representing standard deviations. The statistical significance was assessed by two tailed t-Test (Supplementary Data 1).

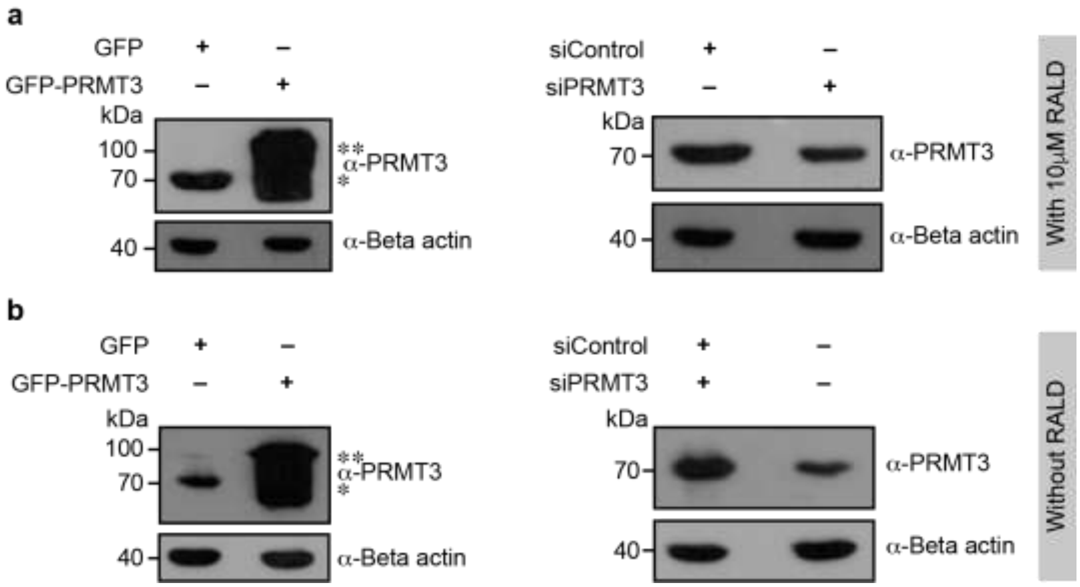

Supplementary Fig. 8. Efficiency of PRMT3 perturbation in HEK293 cells.

HEK293 cells were transfected with GFP vector or GFP-tagged PRMT3 construct or control siRNA or PRMT3 siRNA and treated with (panel **a**) or without (panel **b**) 10 µM of all trans-retinal (RALD). The efficiency of PRMT3 overexpression and knockdown was quantified by immunoblotting with PRMT3 antibody. ** indicates the GFP tagged PRMT3 and * indicates the endogenous PRMT3 (Supplementary Fig. 10).

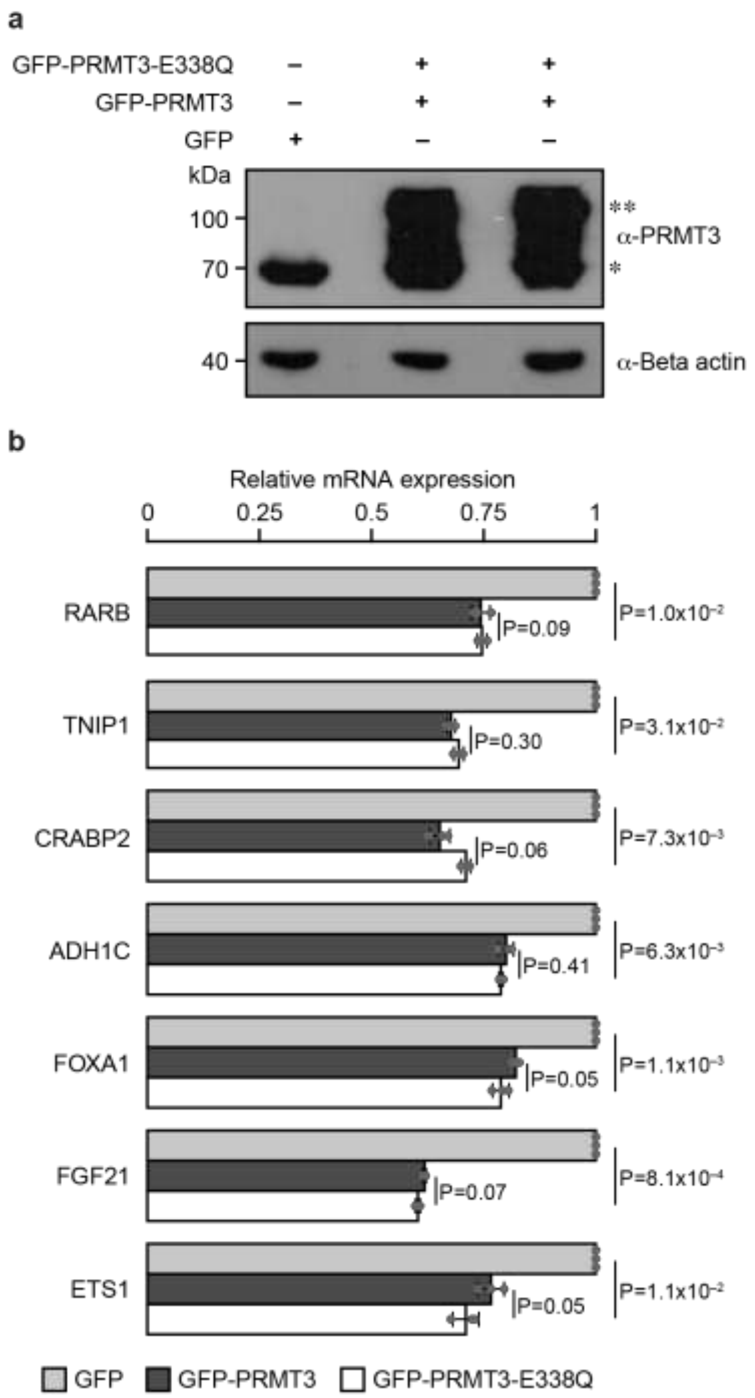

Supplementary Fig. 9. Both wild type PRMT3 and catalytically inactive mutant PRMT3-E338Q decreases the expression of RA responsive genes to a similar extent

HEK293 cells were transfected with GFP vector or GFP-PRMT3 construct or GFP-PRMT3-E338Q construct and treated with 10 µM of all trans-retinal (RALD). (**a**) The efficiency of PRMT3 or PRMT3-E338Q overexpression was quantified by immunoblotting with PRMT3

antibody. ** indicates the GFP-PRMT3 or GFP-PRMT3-E338Q and * indicates the endogenous PRMT3 (Supplementary Fig. 10). (**b**) The graph presents the mRNA levels of the indicated RA responsive genes as relative expression quantified by quantitative RT-PCR. The values in the graphs represent the mean of three independent experiments, with error bars representing standard deviations. The statistical significance was assessed by two tailed t-Test (Supplementary Data 1).

Supplementary Fig. 10

Figure 1b Forward Co-IP

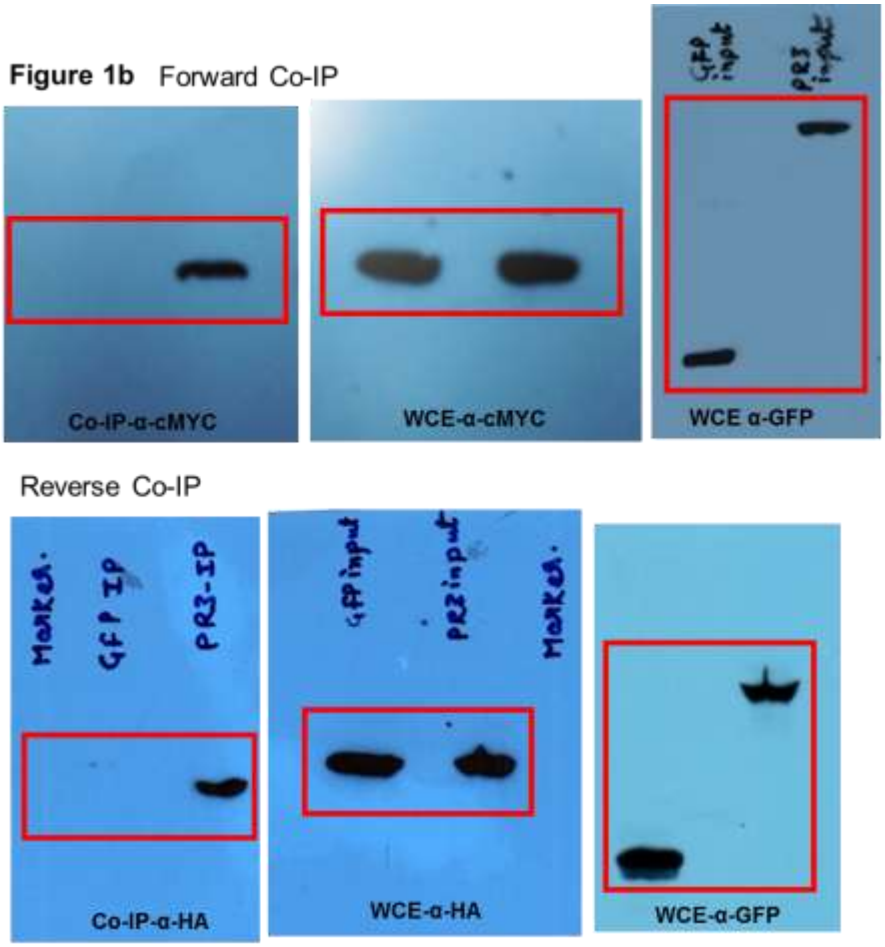

Figure 1c Endogenous IP

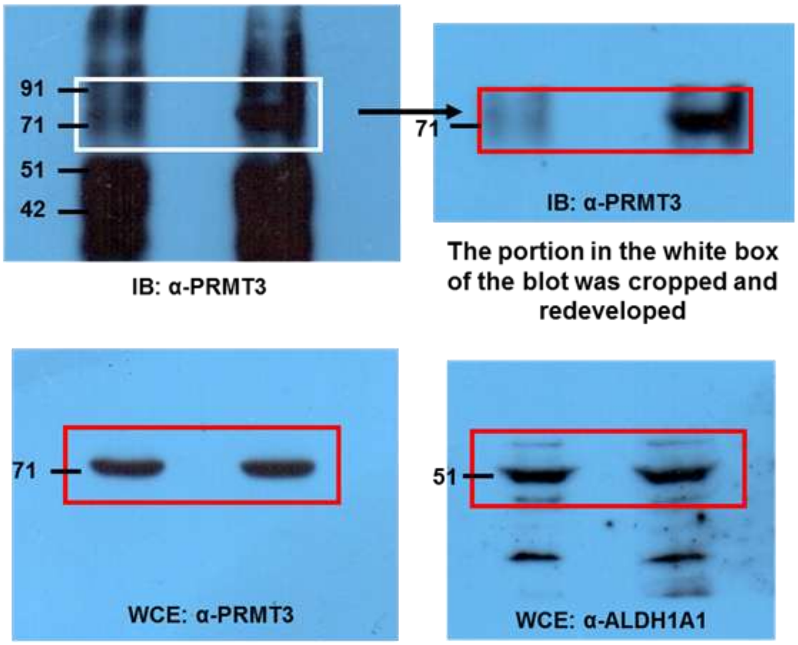

Supplementary Fig. 10 Continued

Figure 1d GST pull down

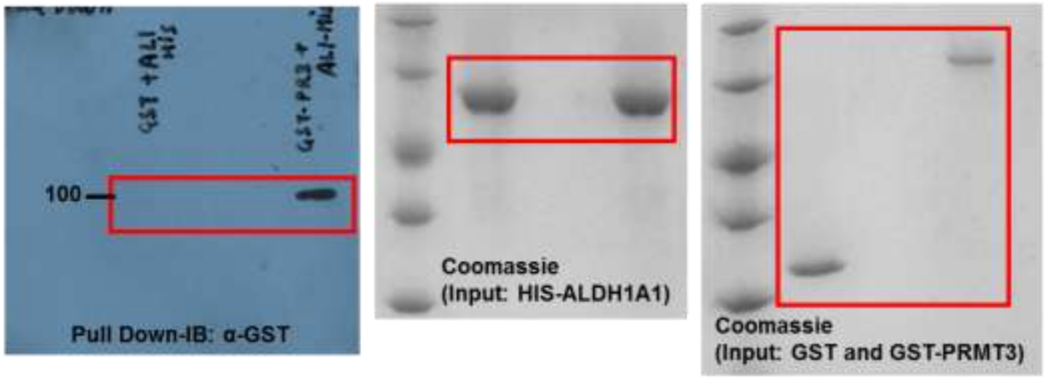

Figure 1e Ni-NTA pull down

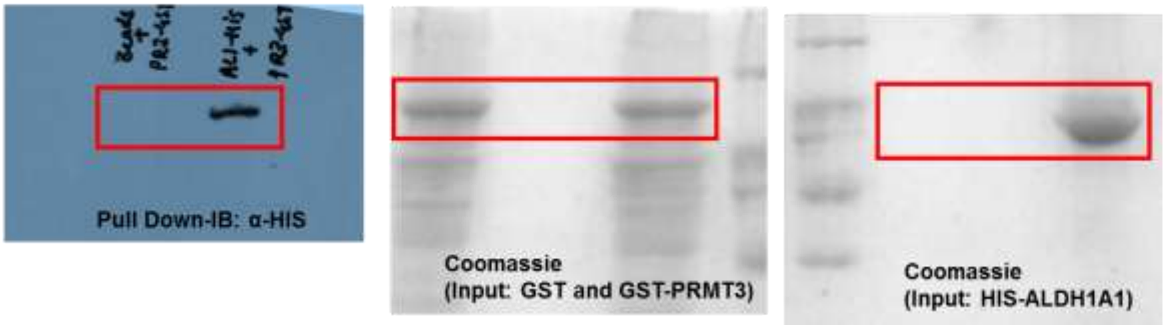

Supplementary Fig. 10 Continued

Figure 1f

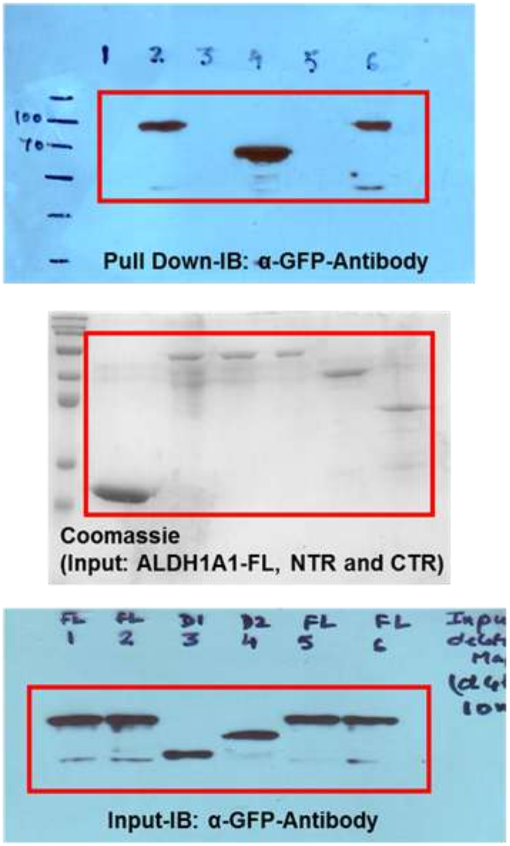

Figure 1g

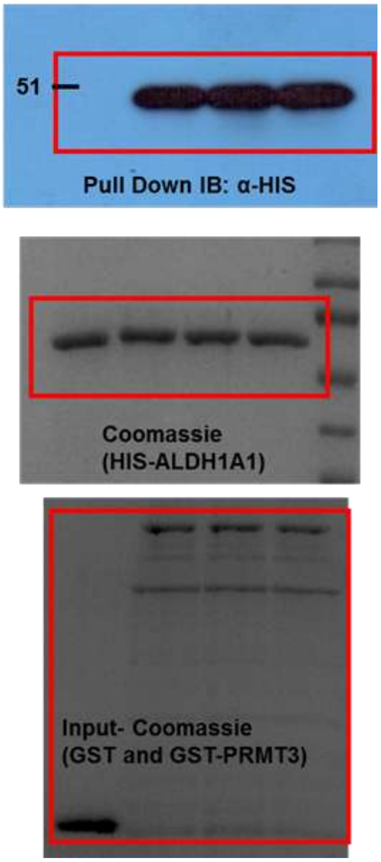

Supplementary Fig. 10 Continued

Figure 2b

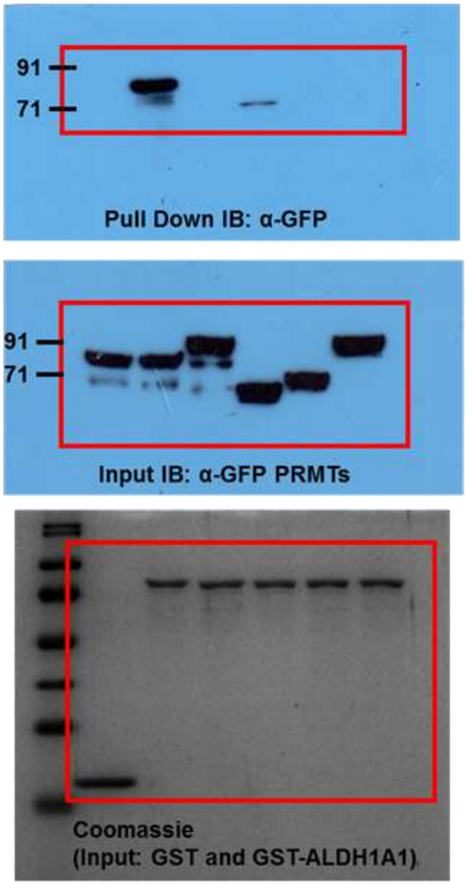

Figure 2d

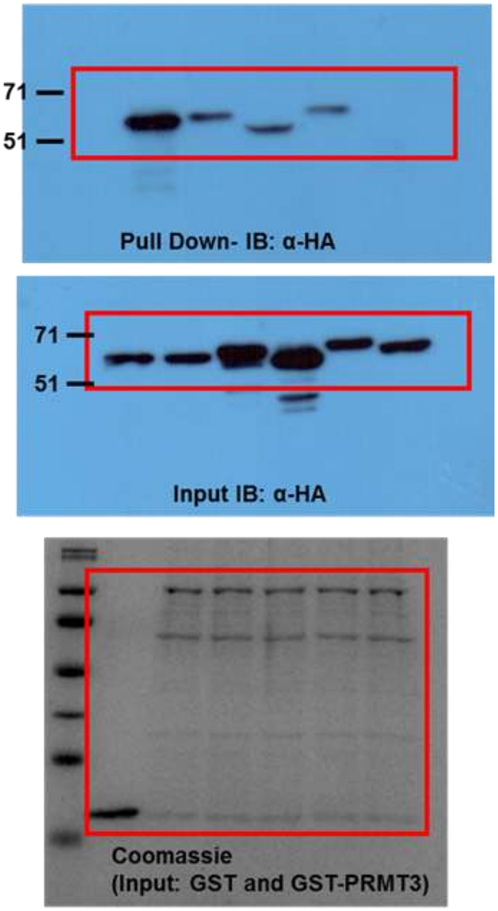

Figure 3c

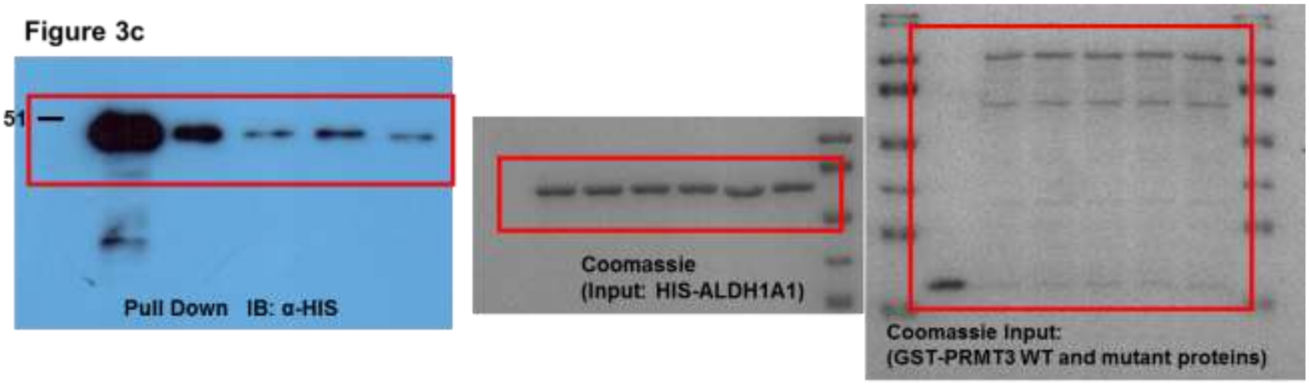

Supplementary Fig. 10 Continued

Figure 4a

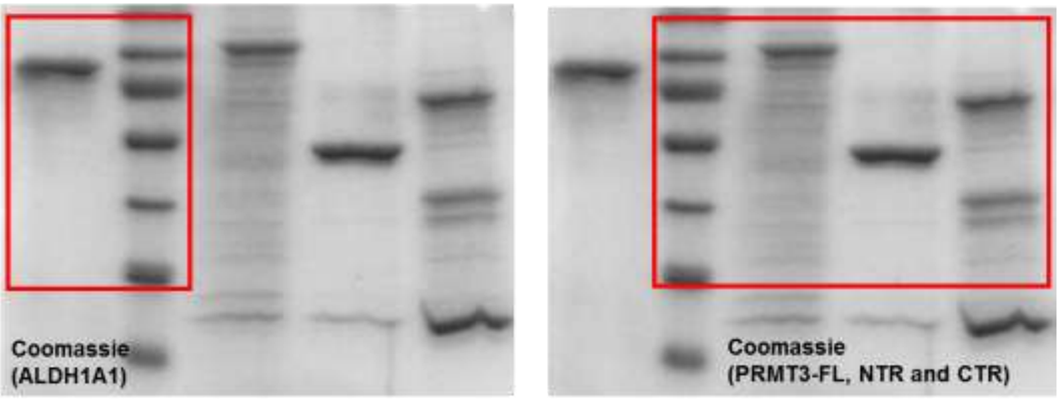

Figure 5a Upper Panel

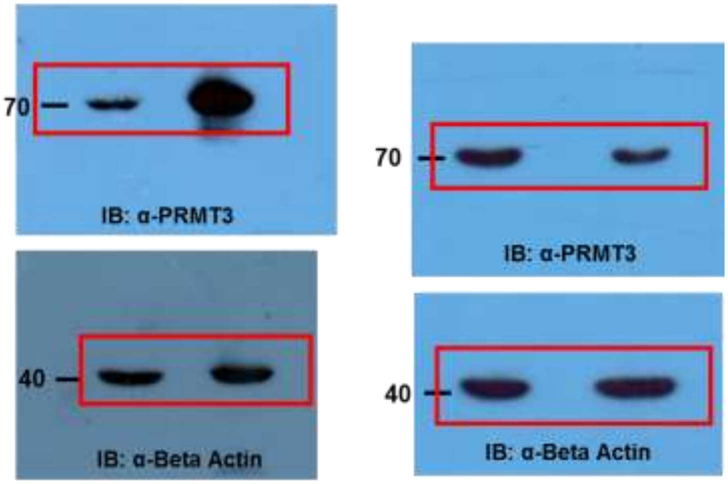

Figure 5b Upper Panel

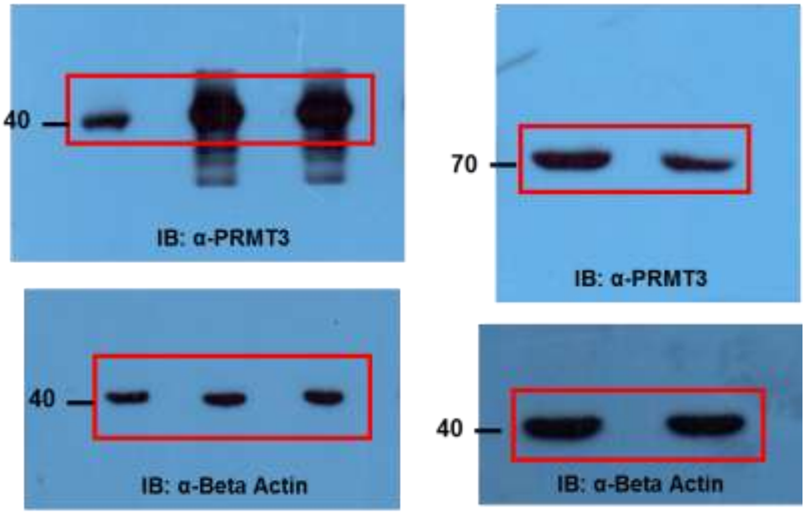

Supplementary Fig. 10 Continued

Supplementary Fig. 4

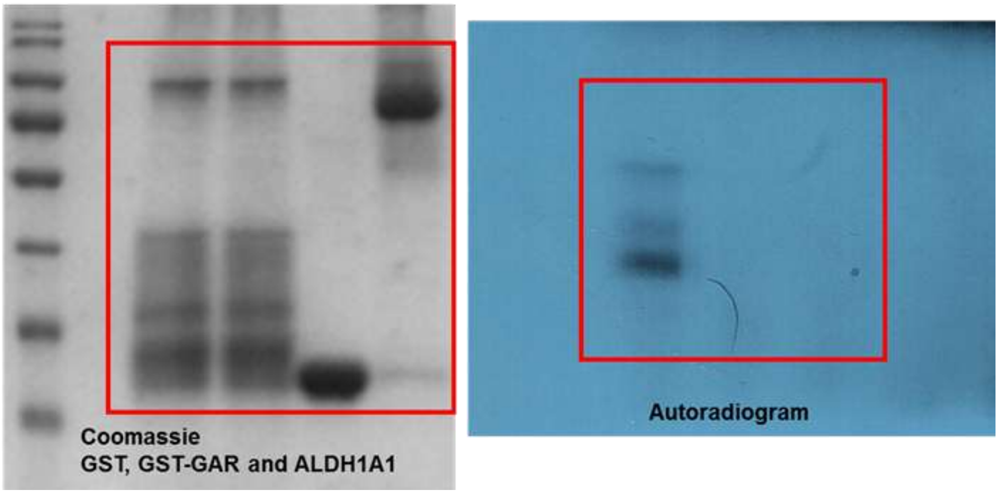

Supplementary Fig. 6b

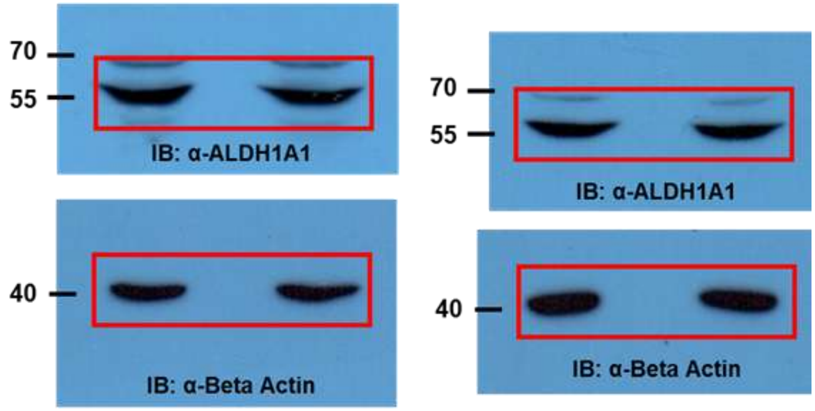

Supplementary Fig. 10 Continued

Supplementary Fig. 8a

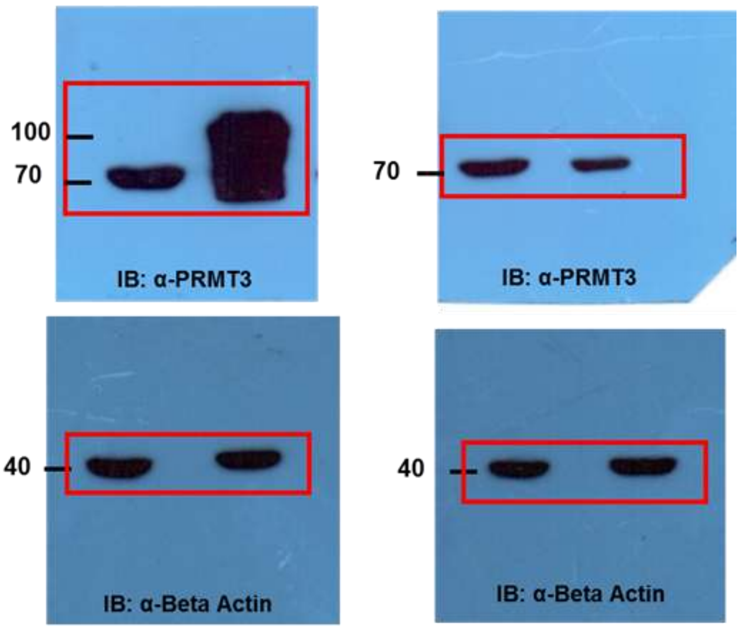

Supplementary Fig. 8b

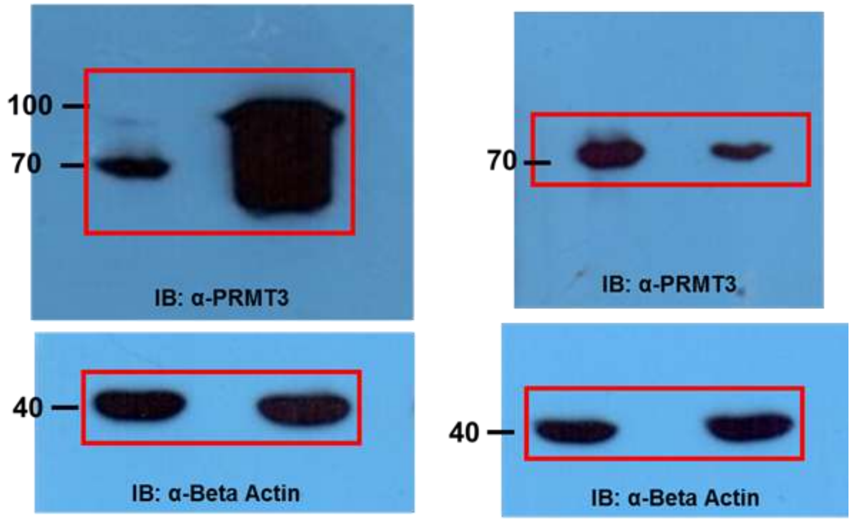

Supplementary Fig. 10 Continued

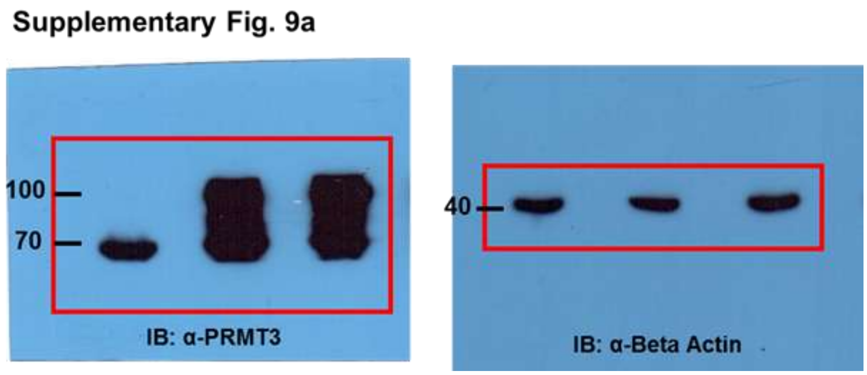

Supplementary Fig. 10. The uncropped immunoblots and gel images of all the figures presented in the main manuscript and supplementary information.

Supplementary Table 1. Protein arginine methyltransferases and Aldehyde dehydrogenases considered in this study (Fig. 2a and Fig. 2c).

Protein	Uniprot accession
**Protein arginine methyltransferases**	
PRMT1	Q99873
PRMT2	P55345
PRMT3	O60678
PRMT4	Q86X55
PRMT5	O14744
PRMT6	Q96LA8
PRMT7	Q9NVM4
PRMT8	Q9NR22
PRMT9	Q6P2P2
**Aldehyde dehydrogenases**	
ALDH1A1	P00352
ALDH1A2	O94788
ALDH1A3	P47895
ALDH1B1	P30837
ALDH1L1	O75891
ALDH1L2	Q3SY69
ALDH2	P05091
ALDH3A1	P30838

**Protein**	**Uniprot accession**
ALDH3A2	P51648
ALDH3B1	P43353
ALDH3B2	P48448
ALDH4A1	P30038
ALDH5A1	P51649
ALDH6A1	Q02252
ALDH7A1	P49419
ALDH8A1	Q9H2A2
ALDH9A1	P49189
ALDH16A1	Q8IZ83
ALDH18A1	P54886

Supplementary Table 2: Functional attributes of Retinoic Acid targets investigated in this study (Fig. 6).

**Gene**	**Function**	**Localisation**	**Biological process**
RARA	Plays role in developmental processes, cell differentiation, apoptosis, granulopoeisis, and circadian rhythm control	Nuclear retinoic acid receptor	Development and Differentiation
RARB	Cellular differentiation during embryonic development	Nuclear transcriptional receptor	Development and Differentiation
FOXA1	Involved in transcription of liver and pancreas genes during development	Nuclear transcription factor	Development and Differentiation
ETS1	stem cell development, cell senescence and death, and tumorigenesis	Nuclear transcription factor	Development and Differentiation
H1F0	Cell differentiation and or proliferation	Nuclear transcription factor	Development and Differentiation
HOXB1	Involved in morphogenesis during development	Nuclear transcription factor	Development and Differentiation
TNIP1	Autoimmunity and tissue homeostasis by NFkB activation	Nuclear shuttling protein	Signalling
CRABP2	Facilitates Retinoic acid signalling pathway by promoting RA binding to receptor	Cytosol to Nuclear shuttling protein	Signalling

Gene	Function	Localisation	Biological process
CD38	Synthesizes the second messengers cyclic ADP-ribose and nicotinate-adenine dinucleotide phosphate, immune receptor	Transmembrane glycoprotein	Signalling
TMG2	Signal transduction	Transmembrane protein	Signalling
HSD17B1	Plays role in estrogen activation and androgen inactivation	Cytoplasmic enzyme	Signalling
ADH3	Involved in metabolism of corticosteroids, biogenic amines, neurotransmitters, and lipid peroxidation	Mitochondrial and nuclear enzyme	Metabolism
FGF21	Stimulated metabolic activity by uptake of glucose, promotes mitogenic and cell survival activities	Secreted extracellularly	Metabolism

Supplementary Table 3: Primers used in qRT-PCR analysis.

Gene	Forward Primer (5′-3′)	Reverse Primer (5′-3′)
GAPDH	TCACCAGGGCTGCTTTTAAC	TGACGGTGCCATGGAATTTG
PRMT3	GGCTTCAAGATGTCCTGCAT	GACCCTGTTGTGGCAATTCT
RARA	CACATGTTCCCCAAGATGCT	GCCCTCTGAGTTCTCCAACA
RARB	GAAAAAGACGACCCAGCAAG	ATGAGAGGTGGCATTGATCC
FOXA1	GAAGATGGAAGGGCATGAAA	GCCTGAGTTCATGTTGCTGA
ETS1	CCAATCCAGCTATGGCAGTT	TTCCTCTTTCCCCATCTCCT
H1F0	GGTCAAGAAGGCCAAGAAGA	GGACTTTGCTTTGGGTTTCA
HOXB1	CTCCGAGGACAAGGAAACAC	AGCTGCCTTGTGGTGAAGTT
TNIP1	CACGCAGAATGAGTTGCTGA	CCTTCTCCTCATTCATGCGC
CRABP2	TGAGGAGCAGACTGTGGATG	TTCAGGAGCTTCTGCTCACA
CD38	GGAGAAAGGACTGCAGCAAC	CCATTGAGCATCACATGGAC
TGM2	GCCTGACTGAGGAGCAGAAG	TCGAAGTTCACCACCAGCTT
HSD17B1	CACAGCAAGCAAGTCTTTCG	CATGGCGGTGACGTAGTTG
FGF21	GAGATCAGGGAGGATGGGAC	CCTGGATGTCTTGACTCCCA
ADH3	CGTTTGAAGTCATCGGTCGG	AATAGCTCCTTTCCACGTGC

Supplementary References

1. Altenhoff, A. M. *et al.* The OMA orthology database in 2015: function predictions, better plant support, synteny view and other improvements. *Nucleic Acids Res.* **43**, D240–D249 (2015).
2. Lin, J. Divergence measures based on the Shannon entropy. *IEEE Trans. Inf. Theory* **37**, 145–151 (1991).
3. Johansson, F. & Toh, H. A comparative study of conservation and variation scores. *BMC Bioinformatics* **11**, 388 (2010).
4. Capra, J. A. & Singh, M. Predicting functionally important residues from sequence conservation. *Bioinformatics* **23**, 1875–1882 (2007).
