# Supplementary material for: PRMT3 interacts with ALDH1A1 and regulates gene-expression by inhibiting retinoic acid signaling
Source: Commun Biol. 2021 Jan 25;4:109. doi: 10.1038/s42003-020-01644-3 (PMC7835222; doi:10.1038/s42003-020-01644-3)
Supplement: Supplementary file 3 — Description of Additional Supplementary Files [file 42003_2020_1644_MOESM3_ESM.pdf]

Description of Additional Supplementary Files

File Name: Supplementary Data 1
Description: Source data for all graphs
