# Supplementary material for: PRMT3 interacts with ALDH1A1 and regulates gene-expression by inhibiting retinoic acid signaling
Source: Commun Biol. 2021 Jan 25;4:109. doi: 10.1038/s42003-020-01644-3 (PMC7835222; doi:10.1038/s42003-020-01644-3)
Supplement: Supplementary file 5 — Reporting Summary [file 42003_2020_1644_MOESM5_ESM.pdf]

nature research

Corresponding author(s): Sreenivas Chavali and Arunkumar Dhayalan

Last updated by author(s): Nov 24, 2020

Reporting Summary

Nature Research wishes to improve the reproducibility of the work that we publish. This form provides structure for consistency and transparency in reporting. For further information on Nature Research policies, see Authors & Referees and the Editorial Policy Checklist .

Statistics

For all statistical analyses, confirm that the following items are present in the figure legend, table legend, main text, or Methods section.

n/a	Confirmed	
☐	☒	The exact sample size (*n*) for each experimental group/condition, given as a discrete number and unit of measurement
☐	☒	A statement on whether measurements were taken from distinct samples or whether the same sample was measured repeatedly
☐	☒	The statistical test(s) used AND whether they are one- or two-sided<br>*Only common tests should be described solely by name; describe more complex techniques in the Methods section.*
☒	☐	A description of all covariates tested
☒	☐	A description of any assumptions or corrections, such as tests of normality and adjustment for multiple comparisons
☐	☒	A full description of the statistical parameters including central tendency (e.g. means) or other basic estimates (e.g. regression coefficient) AND variation (e.g. standard deviation) or associated estimates of uncertainty (e.g. confidence intervals)
☐	☒	For null hypothesis testing, the test statistic (e.g. *F*, *t*, *r*) with confidence intervals, effect sizes, degrees of freedom and *P* value noted<br>*Give P values as exact values whenever suitable.*
☒	☐	For Bayesian analysis, information on the choice of priors and Markov chain Monte Carlo settings
☒	☐	For hierarchical and complex designs, identification of the appropriate level for tests and full reporting of outcomes
☒	☐	Estimates of effect sizes (e.g. Cohen's *d*, Pearson's *r*), indicating how they were calculated

Our web collection on statistics for biologists contains articles on many of the points above.

Software and code

Policy information about availability of computer code

Data collection	Immunoblots were developed using ECL Super signal West Pico Plus Chemiluminescent substrate (ThermoFisher Scientific). The enzymatic activity of ALDH1A1 was quantified by measuring the OD at 340 nm using Spectramax multimode reader (Series: M2E). The Aldefluor assay was performed using ALDEFLUOR Kit, Stemcell Technologies and the fluorescence signal of the cells was quantified in FACS AriaIII flow cytometer (BD Biosciences, USA) using the software FACS Diva version 8. Firefly and Renilla luciferase assays were performed by using Dual-Luciferase Reporter assay kit (Promega) and luminescence signals were measured in Luminometer (Berthold Technologies, Centrol LB.960) using the software MikroWin2010. Quantitative RT-PCR analysis was performed on Light cycler real time PCR system (Roche) using Fast start essential DNA green master mix (Roche).
Data analysis	All data analysis, null hypothesis testing and drawing plots were done using R.

For manuscripts utilizing custom algorithms or software that are central to the research but not yet described in published literature, software must be made available to editors/reviewers. We strongly encourage code deposition in a community repository (e.g. GitHub). See the Nature Research guidelines for submitting code & software for further information.

Data

Policy information about availability of data

All manuscripts must include a data availability statement. This statement should provide the following information, where applicable:

- Accession codes, unique identifiers, or web links for publicly available datasets
- A list of figures that have associated raw data
- A description of any restrictions on data availability

All the data are available from the corresponding authors on request. No restrictions put on the data availability.

Field-specific reporting

Please select the one below that is the best fit for your research. If you are not sure, read the appropriate sections before making your selection.

[x] Life sciences [ ] Behavioural & social sciences [ ] Ecological, evolutionary & environmental sciences

For a reference copy of the document with all sections, see nature.com/documents/nr-reporting-summary-flat.pdf

Life sciences study design

All studies must disclose on these points even when the disclosure is negative.

Sample size	No sample size was predetermined. Three or more independent samples were used to obtain results which were then used to perform statistical analysis. All sample sizes and the number of independent replicates are stated in figure legends.
Data exclusions	None
Replication	The number of independent biological replicates are described in figure legends.
Randomization	No randomization was used.
Blinding	Blinding was not performed since it is not necessary for our study.

Reporting for specific materials, systems and methods

We require information from authors about some types of materials, experimental systems and methods used in many studies. Here, indicate whether each material, system or method listed is relevant to your study. If you are not sure if a list item applies to your research, read the appropriate section before selecting a response.

Materials & experimental systems

n/a	Involved in the study	
[ ]	[x]	Antibodies
[ ]	[x]	Eukaryotic cell lines
[x]	[ ]	Palaeontology
[x]	[ ]	Animals and other organisms
[x]	[ ]	Human research participants
[x]	[ ]	Clinical data

Methods

n/a	Involved in the study	
[x]	[ ]	ChIP-seq
[ ]	[x]	Flow cytometry
[x]	[ ]	MRI-based neuroimaging

Antibodies

Antibodies used

Primary antibodies
ALDH1A1 antibody (Proteintech, Cat. No. 22109-1-AP, 1 in 200 dilution)
PRMT3 monoclonal antibody (Abcam, Cat. No. ab191562, 1 in 3000 dilution)
GFP antibody (Clontech, Cat. No. 632381, 1 in 3000 dilution)
GST antibody (GE Healthcare, Cat. No. 27457701V, 1 in 2000 dilution)
Myc antibody (SCBT, Cat. No. sc-40, 1 in 250 dilution)
His antibody (GE Healthcare, Cat. No. 27-4710-01, 1 in 3000 dilution)
Beta Actin antibody (Sigma, Cat. No. A2228, 1 in 4000 dilution)
Rabbit IgG control antibody (CST, Cat. No. 2729)
HA antibody (Roche, Cat. No. 11867423001, 1 in 1000 dilution)

Secondary antibodies
Anti-mouse HRP conjugated secondary antibody (GE Healthcare, Cat. No. NA931V, 1 in 5000 dilution)
Anti-goat HRP conjugated secondary antibody (SCBT, Cat. No. sc-2020, 1 in 5000 dilution)
Anti-rabbit HRP conjugated secondary antibody (Sigma, Cat. No. A0545, 1 in 4000 dilution)
Anti-rabbit light chain specific HRP conjugated secondary antibody (Millipore, Cat. No. MAB201P, 1 in 3000 dilution)
Anti-rat HRP conjugated secondary antibody (Thermo Scientific, Cat. No. 31470, 1 in 3000 dilution)

Validation

Antibodies were validated as noted on manufacturer's website

Eukaryotic cell lines

Policy information about cell lines

Cell line source(s)	HEK293 cells were purchased from National Centre for Cell Science (NCCS), Pune, India.
Authentication	Cell lines were authenticated by the supplier.
Mycoplasma contamination	Cell culture facility were tested regularly and it was found to be Mycoplasma negative.
Commonly misidentified lines (See ICLAC register)	The cell line used in the study was not a commonly misidentified cell line.

Flow Cytometry

Plots

Confirm that:

- [x] The axis labels state the marker and fluorochrome used (e.g. CD4-FITC).
- [x] The axis scales are clearly visible. Include numbers along axes only for bottom left plot of group (a 'group' is an analysis of identical markers).
- [x] All plots are contour plots with outliers or pseudocolor plots.
- [x] A numerical value for number of cells or percentage (with statistics) is provided.

Methodology

Sample preparation	The details were provided in the methods section of the manuscript.
Instrument	FACS Aria III (BD Biosciences, USA)
Software	FACS Diva version 8
Cell population abundance	Sorting was not performed in this study.
Gating strategy	Only FSC/SSC gates were used for potential live cells and to exclude cell debris.

- [ ] Tick this box to confirm that a figure exemplifying the gating strategy is provided in the Supplementary Information.
